# The gut bacterial microbiome of Nile tilapia (*Oreochromis niloticus*) from lakes across an altitudinal gradient

**DOI:** 10.1186/s12866-022-02496-z

**Published:** 2022-04-04

**Authors:** Negash Kabtimer Bereded, Getachew Beneberu Abebe, Solomon Workneh Fanta, Manuel Curto, Herwig Waidbacher, Harald Meimberg, Konrad J. Domig

**Affiliations:** 1 University of Natural Resources and Life Sciences, Vienna, Austria; 2 Department of Food Science and Technology, Institute of Food Science, Muthgasse 18, 1190 Vienna, Austria; 3 Department of Biology, Bahir Dar University, Post Code 79, Bahir Dar, Ethiopia; 4 Faculty of Chemical and Food Engineering, Bahir Dar Institute of Technology, Bahir Dar University, Post Code 26, Bahir Dar, Ethiopia; 5 Department of Integrative Biology and Biodiversity Research, Institute for Integrative Nature Conservation Research, Gregor Mendel Strasse 33, 1180 Vienna, Austria; 6 MARE−Marine and Environmental Sciences Centre, Faculdade de Ciências, Universidade de Lisboa, Campo Grande, 1049-001 Lisboa, Portugal; 7 Department of Water, Atmosphere and Environment, Institute of Hydrobiology and Aquatic Ecosystem Management, Gregor Mendel Strasse 33, 1180 Vienna, Austria

**Keywords:** Altitude, Diversity, Fish, Gut microbiota, Lake, 16S rDNA

## Abstract

**Background:**

Microorganisms inhabiting the gut play a significant role in supporting fundamental physiological processes of the host, which contributes to their survival in varied environments. Several studies have shown that altitude affects the composition and diversity of intestinal microbial communities in terrestrial animals. However, little is known about the impact of altitude on the gut microbiota of aquatic animals. The current study examined the variations in the gut microbiota of Nile tilapia (*Oreochromis niloticus*) from four lakes along an altitudinal gradient in Ethiopia by using 16S rDNA Illumina MiSeq high-throughput sequencing.

**Results:**

The results indicated that low-altitude samples typically displayed greater alpha diversity. The results of principal coordinate analysis (PCoA) showed significant differences across samples from different lakes. Firmicutes was the most abundant phylum in the Lake Awassa and Lake Chamo samples whereas Fusobacteriota was the dominant phylum in samples from Lake Hashengie and Lake Tana. The ratio of Firmicutes to Bacteroidota in the high-altitude sample (Lake Hashengie, altitude 2440 m) was much higher than the ratio of Firmicutes to Bacteroidota in the low altitude population (Lake Chamo, altitude 1235 m). We found that the relative abundances of Actinobacteriota, Chloroflexi, Cyanobacteria, and Firmicutes were negatively correlated with altitude, while Fusobacteriota showed a positive association with altitude. Despite variability in the abundance of the gut microbiota across the lakes, some shared bacterial communities were detected.

**Conclusions:**

In summary, this study showed the indirect influence of altitude on gut microbiota. Altitude has the potential to modulate the gut microbiota composition and diversity of Nile tilapia. Future work will be needed to elucidate the functional significance of gut microbiota variations based on the geographical environment.

**Significance and impact of the study:**

Our study determined the composition and diversity of the gut microbiota in Nile tilapia collected from lakes across an altitude gradient. Our findings greatly extend the baseline knowledge of fish gut microbiota in Ethiopian lakes that plays an important role in this species sustainable aquaculture activities and conservation.

**Supplementary Information:**

The online version contains supplementary material available at 10.1186/s12866-022-02496-z.

## Background

Natural environmental conditions may have a notable effect on the organism as well as on overall biological characteristics. Animals are subject to environmentally possible adaptive selection in diverse habitats [1, 2]. Among the most extreme conditions, high-altitude environments are intriguing habitats for a large number of animals. Atmospheric pressure declines significantly with increasing altitude, causing hypobaric hypoxia [3]. These changes can affect the main energy production process by suppressing aerobic metabolism. To adapt to different geographical environments particularly to high altitudes, animals have evolved many fascinating ways of dealing with life in these environments. To overcome harsh environmental conditions, animals inhabiting high-altitude areas evolved morphological, physiological, and genetic adaptations, such as changing body masses, raising metabolic rates, and genetic modification [4–6]. For example, the Lake Titicaca frog (*Telmatobius culeus*) has behavioural, morphological, and physiological adaptations that allow aquatic animals to survive at high altitudes (3812 m) [7]. Moreover, to deal with low temperatures cold-adapted animals have antifreeze proteins [8]. Antifreezing proteins, which include specific proteins, glycopeptides, and peptides, are produced by different organisms for cold adaptation. Antifreezing proteins protect cells and body fluids from freezing by reducing the freezing point of water and inhibiting the growth of ice crystals [9].

In animals, numerous microorganisms inhabit the gut and create an intricate intestinal microbiota. These gut microbes live in a kind of symbiotic association with the host organism. Intestinal microbial communities play a critical role in integrating the main essential functions in the host, for instance, nutrient absorption, hampering the colonization of pathogenic organisms, and maintaining normal mucosal immunity, while hosts provide a living environment for the gut microbiota [10]. The reason for structural differences in intestinal microbial communities is reported to be the strong selection and coevolution of the host and its environment [11]. Some studies have reported that the gut microbiota of animals may support host adaptation to different environments [12, 13]. Ruminants in high altitude environments have a gut microbiome with changes in energy-metabolism-related genes [14]. Due to this adaptation, intestinal digesta from sheep and yaks inhabiting high-altitude environments consist of more methane and volatile fatty acids than their low-altitude relatives [14]. Gut microbial communities have a robust fermentation ability and are equipped with more genes involved in the production of volatile fatty acids [14]. From Tibetan and Han populations living at different altitudes, more energy-efficient microbial communities were obtained in samples from those living at higher altitudes [12].

The functions of gut microbial communities depend on their structure, which is influenced by several host-associated elements, such as genetic makeup, season, stress, and geographical location [15–17]. The availability of food resources in different geographical environments is variable and hence significantly affects the diversity and composition of the host gut microbiota [18]. Due to exposure to different geographic environments, the gut microbiota of chickens has changed [13]. Altitude is the main determinant that can modulate the structure and diversity of intestinal microbial communities of animals [12, 19]. Moreover, the impact of high altitude and low oxygen concentration on the gut microbiota of mice was reported recently, which supports the notion of modulation of the gut microbiota composition and metabolic processes due to environmental change [3]. For animals inhabiting aquatic environments at high altitude, low atmospheric pressure, low temperature, and high radiation conditions are some of the factors affecting their physiology [20]. The composition and diversity of fish intestinal microbial communities may be affected by environmental factors such as temperature and salinity, which affect metabolic activity and hence the health status of the host [21].

Nile tilapia (*Oreochromis niloticus*) is a commonly farmed freshwater fish in the world [22]. Nile tilapia is characterized by its capability to endure a broad range of biotic and abiotic stresses, a rapid growth rate, and an omnivorous mode of feeding [23–26]. As a consequence of these qualities, Nile tilapia is an ideal freshwater fish model. Variations in the availability of food resources are the most direct factor modulating the composition of intestinal microbial communities [17]. Nile tilapia feeds on phytoplankton, macrophytes, insects, detritus, and zooplankton [27] depending on its life stages. However, the availability of these food resources differs substantially in several geographical areas [28, 29]. The studies done thus far have emphasized mainly compositional variations and associated adaptive mechanisms of gut microbiota of terrestrial animals inhabiting distinctive geographical locations [3, 30, 31]. However, there is a shortage of studies on the adaptive mechanisms of aquatic animals in water environments. Nile tilapia is a good model for assessing the association of gut microbiota composition and geographical variation due to its adaptation to a multitude of aquatic environments. Investigations on the gut microbial community profile of Nile tilapia inhabiting lakes with distinct altitude environments are greatly lacking. In this study, we aspired to reveal the variations in the gut microbiota profile of Nile tilapia from lakes at distinctive altitudes to provide a deeper understanding of the gut microbiota composition and diversity of the populations of this fish inhabiting differing altitudes. We hypothesized that environmental factor such as altitude would be strongly linked with modulation of the gut microbiota of Nile tilapia. This study contributes to our understanding of the gut microbiota of Nile tilapia from lakes with altitude gradients and provides new insights into this species adaptive mechanisms.

## Results

### Sequencing profiles

The Illumina MiSeq 16S rDNA sequencing data of 39 samples were examined for gut microbiota. After conducting a series of quality filter processes, a total of 88,203 read counts were recovered, with an average of 2261 reads per sample, ranging from 849 to 3357 (Fig. S1). The rarefaction curves had attained a plateau (Fig. S2), suggesting that accurate microbial groups within each sample were demonstrated. Moreover, sequence integrity was assessed using Good’s coverage. The Good’s coverage estimators for all samples in our study were greater than 99, indicating that the majority of bacterial communities in our samples were fully identified. Good’s coverage and alpha diversity indices of the gut bacterial communities are summarized in Table S3.

### Alpha diversity of gut bacterial communities among the studied lakes

**Fig. 1:**
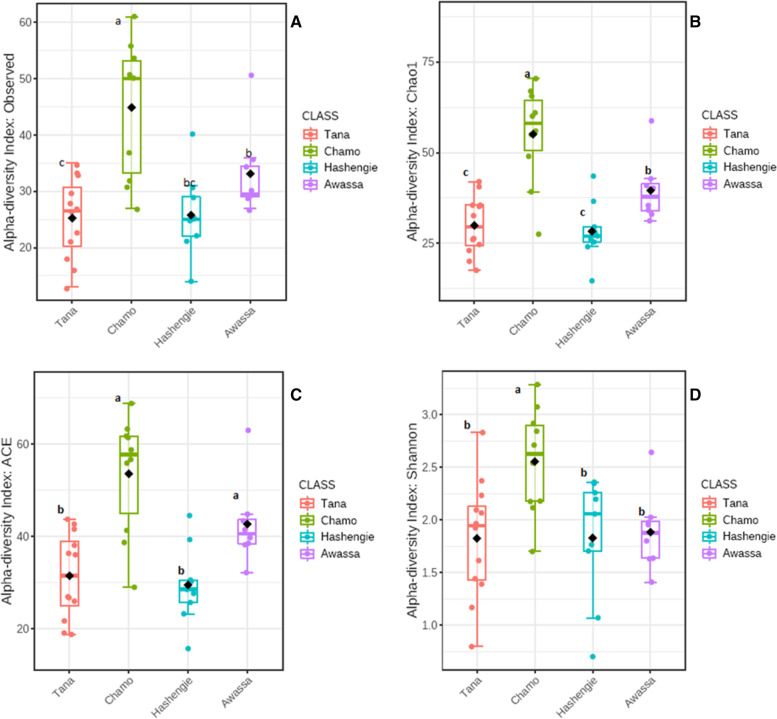
Alpha diversity indices based on sampling lakes. (**A**) Observed index (**B**) Chao1 index (**C**) ACE index (**D**) Shannon index. Independent t- tests was employed to examine the differences between lakes. The significance is shown by small letters a, b, and c.  Boxes with different letters show significant differences (p<0.05)]

Alpha diversity of gut microbiota was examined by Chao1, observed, Accumulated Cyclone Index (ACE), Shannon, Simpson, and Fisher indices (Fig. 1, Fig. S3). Lake Chamo, with a 1235 m altitude, showed the highest value for all indices assessed. Moreover, Lake Chamo samples showed significantly higher alpha diversity than Lake Tana and Lake Hashengie populations in all indices analysed (two-tailed t-test, *p* < *0.05*). The alpha diversity of Lake Awassa was found to be significantly higher than the alpha diversity of Lake Hashengie and Lake Tana, particularly for the Chao1 and ACE indices. Lake Tana and Lake Hashengie showed similar alpha diversity (two-tailed t-test, *p* > *0.05*). The results of the present study suggested that low-altitude samples typically displayed greater alpha diversity.

### Beta diversity of gut microbiota

**Fig. 2:**
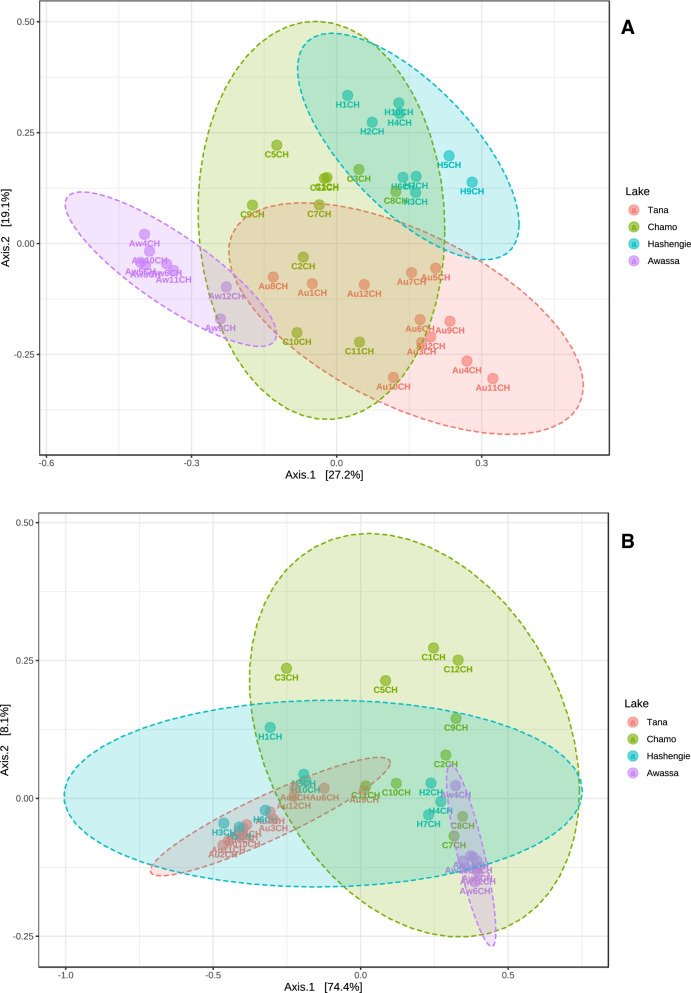
Principal coordinate analysis based on unweighted UniFrac distance (**a**) and weighted UniFrac distance(**b**) of the bacterial community recovered from the gut of Nile tilapia collected from four lakes in Ethiopia located at different altitudes. The explained variances are indicated in brackets

To analyse the intestinal microbiota composition of Nile tilapia from lakes of different altitudes, the beta diversity index was used. To take abundance alteration and phylogenetic association into consideration unweighted UniFrac distance and weighted UniFrac distance were selected as signals of beta diversity. Principal coordinate analysis (PCoA) indicated that substantial variations were observed across samples from different lakes (*p* < 0.001) (Fig. 2). Based on our results, for unweighted UniFrac distance, Axis 1 accounted for 27.2% of the total difference, while Axis 2 accounted for 19.1%, and for weighted UniFrac distance, Axis 1 accounted for 74.4% of the total disparity, while Axis 2 rated 8.1%. We performed an analysis of similarity (ANOSIM) on both unweighted and weighted UniFrac distance results to substantiate this dissimilarity. The ANOSIM outcome indicated that there were significant differences between lakes of different altitudes (unweighted R: 0.72842; *p* value < 0.001; weighted R: 0.58415; p value < 0.001). We also carried out a permutational multivariate analysis of variance (PERMANOVA); the PERMANOVA results were concurrent with those of ANOSIM (unweighted R-squared: 0.45044; *p* value < 0.001; weighted R-squared: 0.58502; *p* value < 0.001).

### Bacterial community structure

**Fig. 3:**
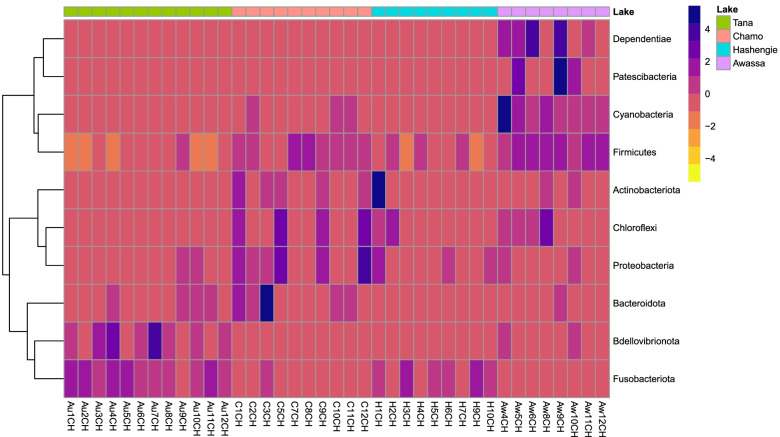
Heatmap showing phyla distribution from the sampling lakes. Clustering was performed based on distance measures using Euclidean and clustering algorithms using Ward at the phylum level

The bacterial phyla recovered from all samples included Bacteroidota (1.8%), Bdellovibrionota (1.2%), Cyanobacteria (0.6%), Firmicutes (52.8%), Fusobacteriota (35.6%), Proteobacteria (6.9%), Chloroflexi (0.3%), Actinobacteriota (0.7%) and Dependentiae (0.1%). However, the proportions of the same bacteria in samples from different lakes were different at the phylum level (Fig. 3, Table 1). There were significant differences in all phyla detected except Bacteroidota and Dependentiae (t-test, two-tailed, *p* value < 0.01). The relative abundance of Bacteroidota in Lake Chamo (0.0434 ± 0.0236) was highest among all groups, followed by Lake Tana (0.0161 ± 0.0048), Lake Awassa (0.0093 ± 0.0085), and Lake Hashengie (0.0004 ± 0.0003). Bdellovibrionota was detected only in Lake Tana and Lake Awassa. The highest Cyanobacteria abundance was recorded in Lake Awassa (0.0211 ± 0.0053). The relative abundance of Firmicutes in the samples of Lake Awassa (0.8941 ± 0.0259) and Lake Chamo (0.6563 ± 0.0670) was much higher than the relative abundance of Firmicutes in the samples from the other two lakes. However, Lake Hashengie and Lake Tana samples showed higher Fusobacteriota abundance than the other lakes. Proteobacteria was highest in Lake Chamo (0.1333 ± 0.0389). Dependentiae was detected only in Lake Awassa (Table 1).

**Table 1:** Relative abundance at the phylum level presented as % (out of 100). The results are expressed as the mean ± standard error of the mean (SEM)

Phyla	Mean ± SEM			
	Lake Awassa	Lake Chamo	Lake Hashengie	Lake Tana
Actinobacteriota	0.75 ± 0.002	1.15 ± 0.004	1.16 ± 0.010	0.01 ± 0.0001
Bacteroidota	0.93 ± 0.009	4.34 ± 0.024	0.04 ± 0.0003	1.61 ± 0.005
Bdellovibrionota	0.74 ± 0.003	0	0	3.53 ± 0.012
Chloroflexi	0.34 ± 0.001	0.54 ± 0.002	0.17 ± 0.001	0
Cyanobacteria	2.11 ± 0.005	0.45 ± 0.002	0	0.12 ± 0.001
Dependentiae	0.25 ± 0.001	0	0	0
Firmicutes	89.41 ± 0.026	65.63 ± 0.067	44.22 ± 0.112	24.24 ± 0.046
Fusobacteriota	1.55 ± 0.008	14.56 ± 0.043	47.52 ± 0.108	66.92 ± 0.056
Proteobacteria	3.86 ± 0.012	13.33 ± 0.039	6.90 ± 0.022	3.58 ± 0.012

The relative abundances of Actinobacteriota, Chloroflexi, Firmicutes, and Fusobacteriota in the Lake Tana samples were significantly different (t-test, two-tailed, *p value* < *0.05*) from the relative abundances of Actinobacteriota, Chloroflexi, Firmicutes, and Fusobacteriota in the samples of Lake Chamo and Lake Awassa. Moreover, Lake Tana samples showed significant variation with Lake Chamo in Bdellovibrionota and Proteobacteria (t-test, two-tailed, *p value* < *0.05*). The ratio of Firmicutes to Bacteroidota in high-altitude populations (Lake Hashengie) was more than the ratio of Firmicutes to Bacteroidota in low-altitude populations (Lake Chamo) by many fold. The Firmicutes to Bacteroidota ratios were found to be 15.13 and 1122.33 in the Lake Chamo and Lake Hashengie samples, respectively.

Investigation of the structure of bacterial communities at the family level resulted in 39 families from all samples (Table S1). Among these families, 27 varied significantly in all samples (two-tailed t-test *p* < *0.05*). The dominant families obtained were Clostridiaceae, Erysipelotrichaceae, Fusobacteriaceae, and Peptostreptococcaceae. The relative abundance of Clostridiaceae in Lake Awassa (0.3930 ± 0.0774) and Lake Chamo (0.2413 ± 0.0381) specimens was greater than the relative abundance of Clostridiaceae in Lake Tana and Lake Hashengie. The relative abundance of Erysipelotrichaceae in Lake Awassa (0.1473 ± 0.0293) was highest among all groups. Lake Tana and Lake Hashengie showed higher Fusobacteriaceae abundances than the two lakes, with relative abundances of 0.6692 ± 0.0563 and 0.4752 ± 0.1075, respectively. The relative abundance of Peptostreptococcaceae in Lake Chamo (0.3901 ± 0.0688) was highest among all groups, followed by Lake Awassa (0.3509 ± 0.0661), Lake Hashengie (0.2540 ± 0.0809), and Lake Tana (0.1447 ± 0.0197). Cyanobiaceae, Microcystaceae, and Rickettsiaceae were not detected in the Lake Hashengie samples. Moreover, some families were detected in only one lake, such as Microbacteriaceae and Solirubrobacteraceae in Lake Hashengie; Microtrichaceae, Ruminococcaceae, and UBA12409 in Lake Awassa; and Silvanigrellaceae in Lake Tana. Five families, namely, Acetobacteraceae, Comamonadaceae, Erysipelotrichaceae, Fusobacteriaceae, and Mycobacteriaceae significantly differed (two-tailed t-test *p* < *0.05*) between Lake Chamo and Lake Hashengie. Fourteen families showed a significant difference between Lake Tana and Lake Awassa, and 16 families showed a significant difference between Lake Tana and Lake Chamo (Table S5).

In total, 27 taxa were identified at the genus level from all samples (Table S2). The majority of these genera (18 genera) varied significantly across sampling lakes at different altitudes (two-tailed t-test *p* < *0.05*). The dominant genera detected were *Cetobacterium*, *Clostridium_sensu_stricto_1*, *Turicibacter,* and *Romboutsia* from nearly all samples. The relative abundance of *Cetobacterium* was found to be higher in Lake Tana (0.6628 ± 0.0580) and Lake Hashengie (0.4750 ± 0.1074) samples than in the other lakes. The relative abundance of *Clostridium_sensu_stricto_1* was highest in Lake Awassa (0.1983 ± 0.0622), followed by Lake Hashengie (0.1388 ± 0.0362)0. Moreover, in Lake Hashengie, which is the lake highest in altitude, the lowest relative abundances of *Hyphomicrobium*, *Macellibacteroides*, *Turicibacter,* and *Uncultured* were obtained compared to the other lakes. In contrast, the relative abundances of *Romboutsia* and *Plesiomonas* were found to be the highest in Lake Hashengie. Some microbial communities were found to be unique for particular lakes only, such as *Silvanigrella* in Lake Tana, *Aurantimicrobium* in Lake Hashengie, *Candidatus_Soleaferrea* in Lake Awassa, and *Nocardioides* in Lake Chamo. *Cetobacterium*, *Nocardioides*, *Turicibacter,* and *Uncultured* significantly differed (two-tailed t-test *p* < *0.05*) between Lake Chamo and Lake Hashengie. The composition of Lake Tana samples was unique since more genera were significantly different (two-tailed t-test *p* < *0.05*) from Lake Awassa (i.e., *Cetobacterium*, *Cyanobium_PCC_6307*, *Microcystis_PCC_7914*, *Romboutsia*, *Silvanigrella*, *Turicibacter,* and *V2*) and Lake Chamo (i.e. *Cetobacterium*, *Nocardioides*, *Romboutsia*, *Silvanigrella*, *Roseomonas,* and *V2*).

### Bacterial signatures in different samples

**Fig. 4:**
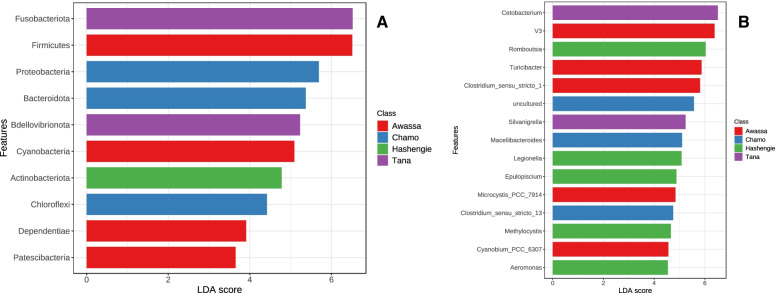
LEfSe results [32]. This figure indicates the microbial communities that were different in abundance between the four sampling lakes. The length of the bar column shows the linear discriminant analysis (LDA) score. (**a**) at phylum level, and (**b**) at genus level

Linear discriminant analysis effect size (LEfSe) was performed to detect the microbial signature in every lake. Signature gut bacterial communities at the genus level comprised *Clostridium_sensu_stricto_1*, *Cyanobium_PCC_6307*, *Microcystis_PCC_7914*, *Turicibacter* and *V3* in the Lake Awassa sample; *Macellibacteroides*, *Clostridium_sensu_stricto_13* and *Uncultured* in the Lake Chamo sample; *Cetobacterium* and *Silvanigrella* in the Lake Tana sample; and *Romboutsia*, *Legionella*, *Epulopiscium*, *Methylocystis* and *Aeromonas* in the Lake Hashengie sample (Fig. 4). At the phylum level, Firmicutes, Cyanobacteria, Dependentiae, and Patescibacteria in Lake Awassa; Proteobacteria, Chloroflexi, and Bacteroidota in Lake Chamo; Fusobacteriota and Bdellovibrionota in Lake Tana; and Actinobacteriota in Lake Hashengie were found to be important taxa.

### Unique and shared bacteria in the gut of Nile tilapia

**Fig. 5:**
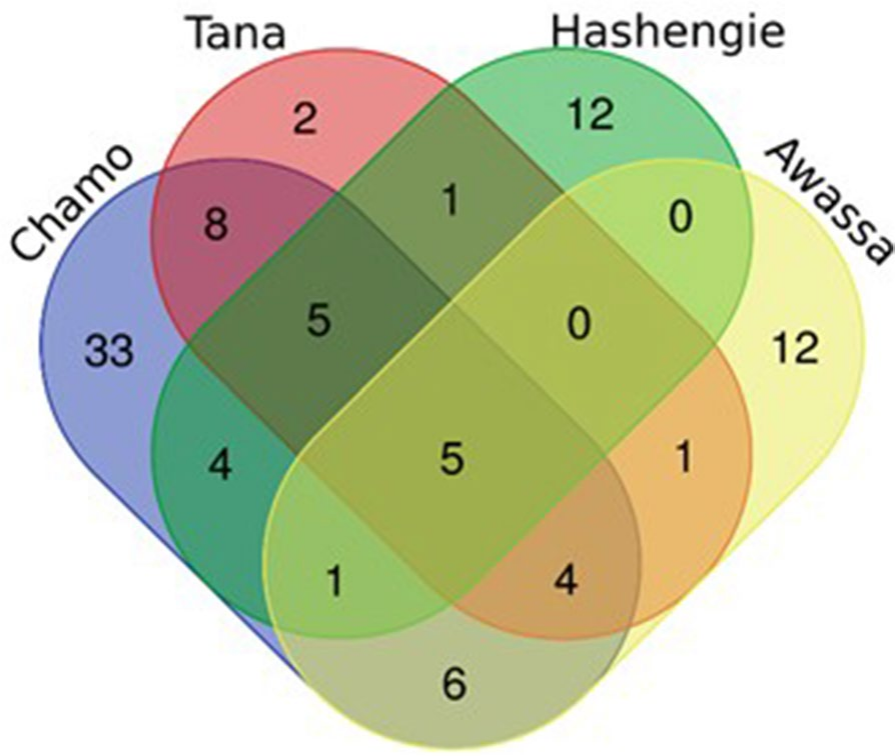
Venn diagram showing the total number of unique and shared ASV numbers in the four sampling lakes

A Venn diagram was made to assess the distribution of amplicon sequence variants (ASVs) among different samples collected from lakes located at different altitudes. The results showed that five ASVs (ASV13, ASV16, ASV2, ASV1, and ASV3) were shared by all lakes. ASV12, ASV110, ASV81, and ASV133 were shared between Lake Hashengie and Lake Chamo. Moreover, six ASVs (ASV47, ASV145, ASV113, ASV44, ASV7, and ASV55) were shared by Lake Awassa and Chamo. However, some ASVs were peculiar to some lakes only, e.g., 12 ASVs in Lake Hashengie, two ASVs (ASV19 and ASV78) in Lake Tana, 12 ASVs in Lake Awassa, and 33 ASVs in Lake Chamo (Fig. 5).

### Correlation between gut microbiota and altitude

To determine which bacterial communities were associated with altitude, Spearman correlation analysis was employed. We found that the relative abundances of Actinobacteriota (Spearman correlation 0.388, *p* value < 0.05), Chloroflexi (Spearman correlation 0.396, *p* value < 0.05), Cyanobacteria (Spearman correlation 0.503, *p* value < 0.01) and Firmicutes (Spearman correlation 0.464, *p* value < 0.01) were negatively correlated with altitude, while Fusobacteriota (Spearman correlation 0.561, p value < 0.01) showed a positive association with altitude (Table 2). At the genus level, altitude was positively correlated with *Aurantimicrobium*, *Legionella,* and *Cetobacterium*. However, 11 genera, *Clostridium_sensu_stricto_13*, *Hyphomicrobium*, *Macellibacteroides*, *Methyloparacoccus*, *Microcystis_PCC_7914*, *V2*, *Nocardioides*, *Roseomonas*, *Shewanella*, *uncultured* and *Turicibacter*, showed negative correlations with altitude (Table S4).

**Table 2:** Spearman correlation between the relative abundances of gut bacterial communities at the phylum level and altitude. ^a^Correlation is significant at the 0.01 level (2-tailed). ^b^Correlation is significant at the 0.05 level (2-tailed)

Taxa	Correlation Coefficient	Sig. (2-tailed)
Actinobacteriota	-0.388^b^	0.015
Bacteroidota	-0.295	0.068
Bdellovibrionota	0.068	0.681
Chloroflexi	-0.396^b^	0.013
Cyanobacteria	-0.503^a^	0.001
Dependentiae	-0.215	0.189
Firmicutes	-0.464^a^	0.003
Fusobacteriota	0.561^a^	0.000
Others	-0.171	0.298
Proteobacteria	-0.103	0.533

## Discussion

Various studies have shown that the composition of the gut microbiota of animals is affected by altitude [3, 30, 31, 33]. Animals inhabiting high-altitude environments are characterized by unique gut microbiota compared to those living at low altitudes. There is a shortage of investigations on the intestinal microbial communities of fish species from lakes located at different altitudes. Our study investigated the effect of altitude on gut-associated microbial communities of Nile tilapia from four Ethiopian lakes located at different altitudes. This study included Lake Hashengie, a high-altitude lake in Ethiopia, and this is the first investigation of the intestinal bacterial communities of Nile tilapia in Lake Hashengie using high-throughput sequencing. In our study, the question of how the gut bacterial communities were affected with respect to altitude was addressed.

From the studied lakes, Nile tilapia are not native to Lake Hashengie, the lake located at an altitude of 2440 m. The Nile tilapia in Lake Hashengie are believed to have been introduced and adapted to the high-altitude environment [34]. At high altitudes aquatic organisms face physiological challenges as a response to lower levels of nutrients, buffering capacity, atmospheric pressure, and temperatures [20]. Moreover, high-altitude lakes are characterized by strong ultraviolet radiation and low primary productivity [20, 35]. The low air pressure at high altitudes could affect the amount of dissolved oxygen in lakes and therefore the microbiome community. The decrease in temperature with altitude can have similar effects. A previous study suggested that water temperature may affect the intestinal microbial communities of fishes [36]. The composition and diversity of gut microbiota are reported to take part in a crucial role in host environmental adaptation [33, 37]. In the present investigation, we showed that Nile tilapia inhabiting lakes with dissimilar altitudes have comparatively different gut microbiota and corroborated that altitudinal differences were a determinant that formed the composition of gut bacterial communities in Nile tilapia on Ethiopian lakes. Our findings were consistent with prior studies that showed the effect of altitude on gut microbiota in different animals [3, 30, 31, 33]. In high-altitude mice, hypoxia was found to be one of the key factors that caused modification of the gut microbiota [3]. As reported in high-altitude mice, hypoxia might be the factor for variation of the gut microbiota in the Nile tilapia along the altitude gradient.

Lake Chamo (lowest altitude) samples demonstrated the topmost values on the global alpha-diversity assessments, which were calculated on the rarefied ASVs. The occurrence of a more diversified gut microbiota is believed to be evidence of a robust microbiota [38]. A great alpha diversity attests to a vast number of microbial communities, which may help the host assimilate different diets. Thus, Lake Chamo Nile tilapia samples with enormous diversity may have a greater potential to use a variety of food sources and support fulfillment of their dietary demand in this particular habitat. Beta diversity assessment showed significant differences between gut-related bacterial communities of Nile tilapia. Both the unweighted UniFrac and weighted UniFrac approaches revealed clear significant clustering by the source of the samples in the PCoA plot (Fig. 2), suggesting that altitude may modulate the abundance of different microbial communities in the gut.

In our study, Firmicutes was the most abundant phylum in the Lake Awassa and Lake Chamo samples, whereas Fusobacteriota was the dominant phylum in the samples of Lake Hashengie and Lake Tana. Previous studies on the intestinal microbial communities of Nile tilapia from Ethiopian lakes showed dominance by the phylum Firmicutes followed by Fusobacteriota [15, 39]. Fusobacteriota was also reported as a dominant phylum from the intestine of Nile tilapia of Lake Nasser in Egypt [40] and captive Nile tilapia [41]. Clostridiaceae and Peptostreptococcaceae were the predominant families from samples of Lake Awassa and Chamo, while Fusobacteriaceae was the most dominant in Lake Hashengie and Lake Tana. Ideally, we should have more low- and high-altitude lakes to ensure that the patterns we find are indeed a result of altitude change and not other factors. However, Lake Tana and Lake Hashengie (the lakes located at higher altitudes) show some commonalities that may be evidence that altitude plays a role. In an earlier study, Clostridiaceae played essential roles in carbohydrate degradation in the gut [42]. Peptostreptococcaceae is reported to be involved in the fermentative type of metabolism of proteinaceous substrates and carbohydrates [43]. Importantly, the relative abundances of Fusobacteriaceae, Beijerinckiaceae, Lachnospiraceae, and Vibrionaceae clearly showed higher values at Lake Hashengie than at Lake Chamo, demonstrating that these microorganisms may help the host adapt well to high-altitude environments. To the best of our knowledge, phyla such as Dependentiae, Patescibacteria, and Bdellovibrionota were identified for the first time from the gut of Nile tilapia. These results imply that the intestine of Nile tilapia might harbour more diversified microbial communities in addition to the microbial communities reported thus far. Patescibacteria and Dependentiae dominated the pupfish gut [44]. Patescibacteria is associated with biodegradable plastics such as polylactic acid [45], and has also been reported as a potential microbial bioindicator of phosphorus mining [46]. The phylum Bdellovibrionota has many predatory species, that employ a range of strategies to attack their bacterial prey [47].

The relative abundance of Firmicutes varied significantly among the studied Nile tilapia samples. Firmicutes can produce several enzymes for the degradation of dietary nutrients, thus assisting their hosts in the digestion and absorption of nutrients [48]. Since the gut microbiota of Nile tilapia comprised a large proportion of Firmicutes, we can deduce that the host might be effective in obtaining energy from dietary nutrients. In addition, we also found that the high-altitude samples (Lake Hashengie) had an increased Firmicutes:Bacteroidetes ratio compared with the Firmicutes:Bacteroidetes ratio of the low-altitude samples (Lake Chamo). The higher ratio of Firmicutes to Bacteroidetes in the gut microbiota is reported to be associated with the efficient absorption of food energy [49]. Moreover, the increase in the ratio of Firmicutes to Bacteroidetes was associated with better herbage energy utilization ability and increased resistance to cold stress in the gut of mammals inhabiting high altitudes [30]. In our study, the increase in the ratio of Firmicutes to Bacteroidetes in Lake Hashengie samples indicates that high-altitude Nile tilapia may have efficiency in harvesting energy and may also help them adapt to the environment. In terrestrial animals, consumption of meat and dairy products is associated with a higher Firmicutes:Bacteroidetes ratio and, in contrast, low Firmicutes: Bacteroidetes ratios related to consumption of fruits and vegetables [31, 50, 51]. Lake Hashengie harbours a higher diversity of zooplankton [52]. Moreover, zooplankton were found to be the dominant food source for Nile tilapia in Lake Hashengie [27]. Therefore, the high Firmicutes:Bacteroidetes ratio in Lake Hashengie might help Nile tilapia consume zooplankton. A higher Firmicutes:Bacteroidetes ratio was also associated with the gut of obese animals and humans compared with normal-weight individuals [53, 54]. Fish from Lake Hashengie were extremely fatty, most likely due to the dominance of zooplankton in their food [52] and the high Firmicutes:Bacteroidetes ratio in Lake Hashengie confirmed that the association with obesity also works for Nile tilapia.

Fusobacteriota was found to be positively correlated with altitude, which was principally ascribed to the substantial increasing abundance of the genus *Cetobacterium.* In contrast, a negative correlation between Actinobacteriota, Chloroflexi, Cyanobacteria, and Firmicutes with altitude was observed. Lower microbial taxon richness in high-altitude lakes compared to reference lakes located at lower elevations has been reported previously [35]. In contrast to our study, high-altitude lakes were reported to have a higher abundance of Cyanobacteria [55, 56].

At the genus level, *Cetobacterium*, *Turicibacter,* and *Nocardioides* were revealed as the genera that varied significantly between the low elevation (Lake Chamo) and high elevation (Lake Hashengie) samples. The genus *Cetobacterium,* which was found in the family Fusobacteriaceae, was one of the bacterial genera positively correlated with altitude. Cetobacterium isolated from freshwater fish produces vitamin B-12 [57]. *Turicibacter* species are involved in the modulation of bacterial colonization in the gut, regulation of host energy metabolism, and host immunity [58–60]. *Nocardioides* were detected in the gut of shrimp [61] and a masculinization pond of Nile tilapia fry [62]. *Nocardioides* were reported to be involved in the degradation of steroids and latex [62, 63]. *Romboutsia*, *Legionella*, *Epulopiscium*, *Methylocystis,* and *Aeromonas* are unique and signature gut bacteria in Lake Hashengie, demonstrating that they might perform a crucial function in adaptation to high-altitude habitats.

Despite the variability in the abundance of the gut microbiota across the sampling lakes, a number of shared bacterial communities, including Proteobacteria, Firmicutes, and Fusobacteria, were detected. These microbial communities may be critical for the assembly and function of a gut microbiome, which may highlight their importance to host performance [64]. The shared bacterial communities within the gut of Nile tilapia might be due to the availability of specific microorganisms in the water of the lake that are capable of colonizing the gut and due to selective pressures within the gut habitat.

## Conclusions

In conclusion, the gut microbiota of Nile tilapia from lakes at different altitudes in Ethiopia was successfully characterized by Illumina MiSeq sequencing. Our results showed that the composition and diversity of gut bacterial communities between lakes were different. These variations in intestinal microbiota are possibly due to a consequence of various selection pressures occurring in these habitats, mainly altitude. Our results also highlight the dominance of Firmicutes in the Lake Awassa and Lake Chamo samples and Fusobacteriota in the Lake Hashengie and Lake Tana samples. The ratio of Firmicutes to Bacteroidota in high-altitude samples (Lake Hashengie) was many-fold higher than the ratio of Firmicutes to Bacteroidota in low-altitude populations (Lake Chamo), suggesting similarity with terrestrial animals. This study sheds new light on the associations between altitude and gut microbiota in aquatic animals and provides a perspective on fish ecological adaptation. Furthermore, this study provides valuable information to establish sustainable growth for the aquaculture sector in different geographical locations. Nevertheless, further work needs to be done to better understand how altitude is affecting Nile tilapia’s gut microbiome. First, functional significance of gut microbiota variations based on the geographical environment should be explored in future studies by adding more populations and larger sampling sizes. Second, previous study showed that seasonality influences the microbiota of gut content of Nile tilapia [15]. This effect may be different in different altitudes, which needs to be further explored. Third, the differences found might be a consequence of the microbiome composition of the lakes and not necessarily an adaptation of Nile tilapia. Therefore, a comparison between the microbial communities in the water and the fish gut is needed.

### Methods and materials

#### Description of the sampling sites

**Fig. 6:**
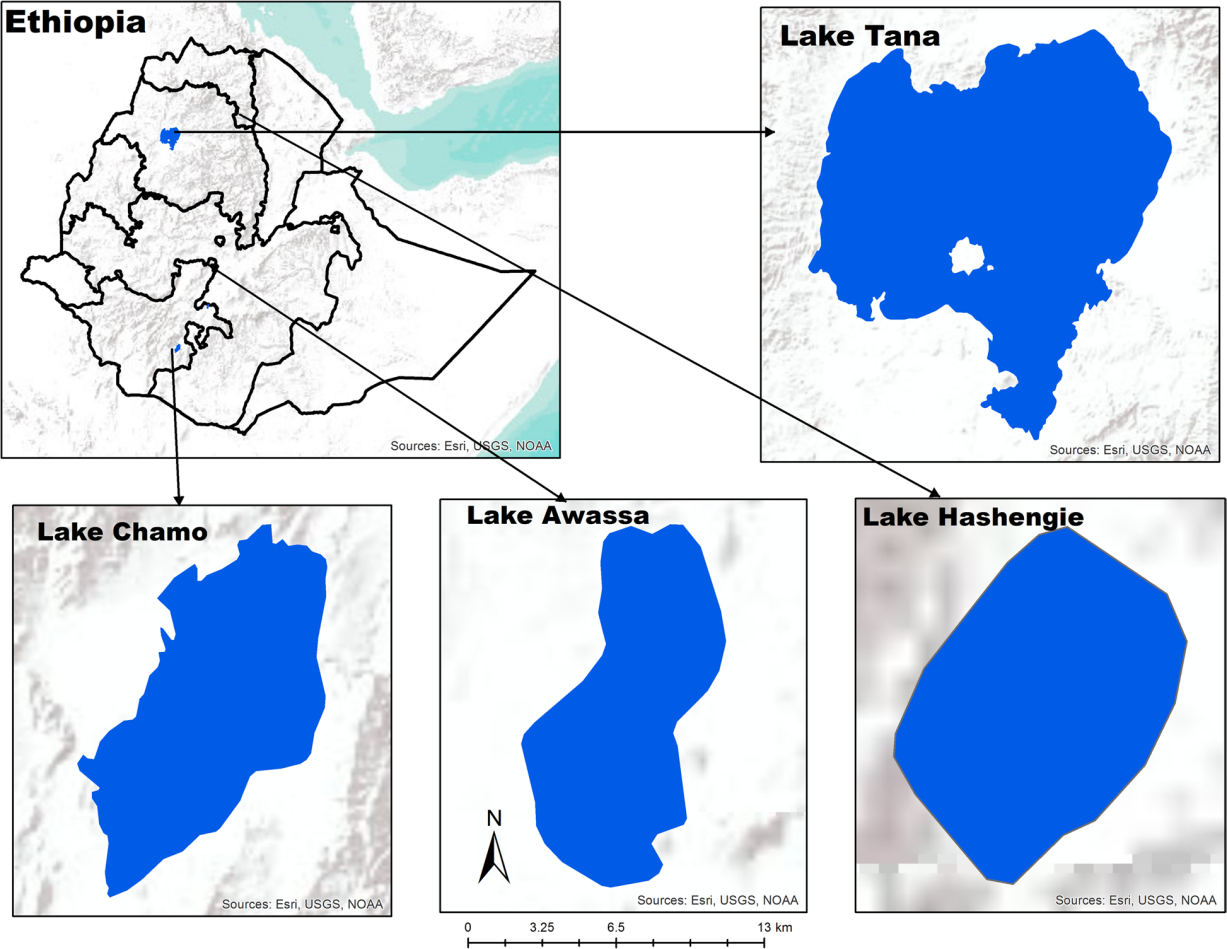
A map showing the location of the four sampling lakes

The samples were collected in July and August 2018 from four lakes with different altitudes, i.e., Lake Awassa, Lake Chamo, Lake Tana, and Lake Hashengie (Fig. 6, Table 3). Lake Awassa and Lake Chamo are located in the Great Rift Valley whereas Lake Tana and Lake Hashengie are not.

**Table 3:** Morphometric characteristics of the four sampling lakes

Lakes	Altitude (m.a.s.l)^a^	Location		Mean depth (m)
		**Latitudes**	**Longitudes**	
Awassa	1685	06^0^58’ to 07^0^14’ N	38^0^22’ to 38^0^28’ E	11
Chamo	1235	5^0^50’ to 5^0^83’ N	37^0^33’ to 37^0^55’ E	10
Tana	1800	10^0^95′ and 12^0^78′ N	36^0^89′ to 38^0^25′ E	8
Hashengie	2440	12^0^31′9`` N	39^0^30′50`` E	14

^a^Meters above sea level

#### Specimen handling and processing

Nile tilapia (*Oreochromis niloticus*) samples were purchased at the landing site of the sampling lakes from the fishermen. Sample collection was performed from June 30, 2018 to August 8, 2018. In this study, only adult male fish samples showing no gross or clinical signs of diseases were included. Males were selected due to their larger size. A total of 39 samples (12 from Lake Tana, 10 from Lake Chamo, 9 from Lake Hashengie and 8 from Lake Awassa) were collected and treated as reported previously [39]. All samples were sacrificed using high doses of clove oil [65]. Ethanol (70%) was applied to the body surface of the fish, and instruments were used for dissection. The intestine was dissected, and the luminal contents from the posterior region were collected in sterile screw-cap tubes filled with phosphate buffered saline and glycerol (50:50, v/v) and stored at -20 °C until further processing [66].

#### Microbial DNA Extraction, PCR Amplification, and Sequencing

Microbial DNA extraction, polymerase chain reaction (PCR) amplification, PCR product purification, and amplicon sequencing processes were performed as previously described [39]. Briefly, genomic DNA extraction was carried out using the PowerFecal® DNA Isolation Kit (Qiagen, Hilden, Germany) according to the manufacturer's instructions with some modifications. The modifications include heating the tubes after addition of C1 solution at 70 °C and using 50 µL warm elution buffer preheated at 40 °C at the end. The Illumina MiSeq paired‐end sequencing system (Illumina, San Diego, CA, USA) was used to sequence the V3-V4 region of the microbial 16S rRNA gene. Two-step PCR was performed to prepare DNA sequencing libraries using the dual index approach following Shokralla et al. [67]. The first PCR was conducted with the primers 347F and 803R from Nossa et al. [68] extended with the Illumina adapter sequences. Briefly, 4 µL of genomic DNA was used as a template for the first PCR (95 °C for 15 min; 30 cycles of 95 °C for 30 s, 55 °C for 1 min, and 72 °C for 1 min; and a final extension at 72 °C for 10 min). For index PCR, TrueSeq adapter sequences were used. The reaction was carried out with the purified PCR product as a template, after an initial denaturation and activation at 95 °C for 15 min, using 10 cycles of 95 °C for 30 s, 58 °C for 60 s, and 72 °C for 60 s. Both PCRs were performed in 10 µL reaction volumes amplifying with 5 µL of QIAGEN Multiplex PCR Master Mix (Qiagen, Hilden, Germany)[39]. The PCR product of each sample was purified by the magnetic bead extraction method. The 16S rRNA genes were sequenced at the Genomics Service Unit, Ludwig‐Maximilian’s‐Universität München, Germany.

#### Analysis of sequence data

Regions with low sequence quality, adapter, and primer sequences were trimmed with Cutadapt v. 0.11.1 software as described previously [39, 69]. Paired-end reads were merged with PEAR v. 0.9.4 [39, 70]. Chimeric sequences were detected with USEARCH 6.0 based on the RDP pipeline [71]. High-quality read sequences were dereplicated using USEARCH and finally denoised to produce amplicon sequence variants (ASVs) using the USEARCH *-*unoise3 command [72]. After mapping the reads for each sample to the list of ASVs, an “OTU” table was made using the ‘otutab’ command in USEARCH. For the classification of the generated ASVs and construction of the phylogenetic tree, the curated SILVA taxonomy was used [73]. Furthermore, to augment the downstream statistical analysis, low‐quality features were removed using minimum counts of 2 and 10% prevalence in samples on MicrobiomeAnalyst [32]. Moreover, ASVs assigned as chloroplasts and mitochondria were removed before downstream analysis. To solve problems of the variability in sampling depth, data rarefaction to a minimum library size was carried out. Moreover, sequence reads were normalized by total sum scaling approaches.

#### Data analysis

Alpha diversity and beta diversity analyses was done using the phyloseq package as implemented in MicrobiomeAnalyst [32]. The alpha diversity of each sample was examined using the Chao1, observed, ACE, Shannon, Simpson, and Fisher indices. For the beta diversity, the dissimilarity matrix was measured using Unweighted Unifrac distance and weighted Unifrac distance method and visualized by principal coordinate analysis (PCoA). Permutational multivariate analysis of variance (PERMANOVA) and analysis of group similarities (ANOSIM) were employed to assess the statistical significance of the clustering pattern in ordination plots. Linear discriminant analysis effect size (LEfSe) were employed to identify the taxa with substantially different relative abundance across all samples [74]. Hierarchical clustering is performed with the function hclust in the package stat [32]. The numbers of shared and unique ASVs are presented in Venn diagrams (http://bioinformatics.psb.ugent.be/webtools/Venn/). Only ASVs present in at least 40% of the samples were included for constructing the Venn diagram. Independent *t-tests* were used to examine the variations in microbial abundance and alpha indices. Spearman's rank correlation test was used to identify which microbial communities were significantly associated with altitude. Statistical analysis was done with SPSS 21 for Windows. All statistical analyses were performed with a significance level of α = 0.05 (*p* < 0.05) unless otherwise stated.

## Supplementary Information

**Additional file 1.** 

**Additional file 2.** 

**Additional file 3.** 

**Additional file 4:** **Supplementary Table 1.** Relative abundance of microbial communities at the family level. Results are expressed as the mean ± standard error of mean (SEM).

**Additional file 5:** **Supplementary Table 2.** Microbial communities identified at genus level from all samples. Results are expressed as the mean ± standard error of mean (SEM).

**Additional file 6:** **Supplementary Table 3.** Alpha diversity indexes and goods coverage of microbial 16S rRNA sequences from the gut of Nile tilapia collected from the four sampling lakes.

**Additional file 7:** **Supplementary Table 4.** Spearman correlation between the relative abundances of gut microbial communities at genus level and altitude. **. Correlation is significant at the 0.01 level (2-tailed). *. Correlation is significant at the 0.05 level (2-tailed).

**Additional file 8:** **Supplementary Table 5.** Microbial communities showing a significant difference at family level between sampling lakes. Comparison was done by independent t-test.

## Data Availability

The raw sequences have been deposited in the National Center for Biotechnology Information (NCBI) Sequence Read Archive (SRA), under the BioProject ID PRJNA763202 accession numbers SRX12193142 to SRX12193150, and some of the samples were found under Bioproject IDs PRJNA705209 and PRJNA637890.

## Abbreviations

ANOSIM: Analysis of group similarities
ASV: Amplicon sequence variant
LEfSe: Linear discriminant analysis effect size
OUT: Operational taxonomic unit
PCoA: Principal coordinate analysis
PCR: Polymerase chain reaction
PERMANOVA: Permutational multivariate analysis of variance
