# Supplementary figures and images for: The gut bacterial microbiome of Nile tilapia (Oreochromis niloticus) from lakes across an altitudinal gradient
Source: BMC Microbiol. 2022 Apr 4;22:87. doi: 10.1186/s12866-022-02496-z (PMC8978401; doi:10.1186/s12866-022-02496-z)

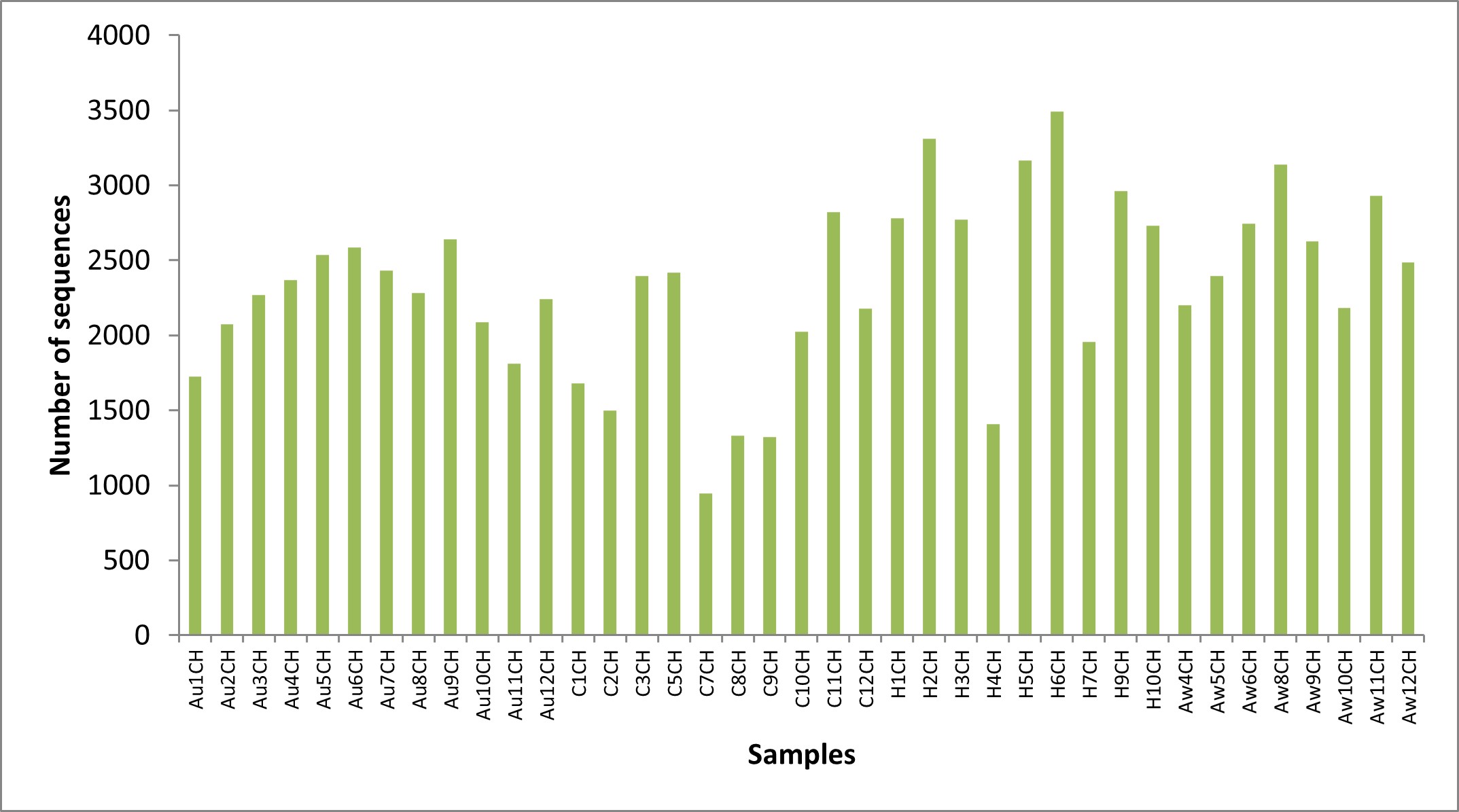

Supplement: Supplementary file 1 — Additional file 1. [file 12866_2022_2496_MOESM1_ESM.jpg]

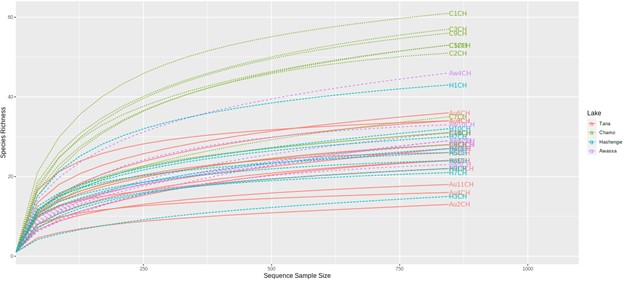

Supplement: Supplementary file 2 — Additional file 2. [file 12866_2022_2496_MOESM2_ESM.jpg]

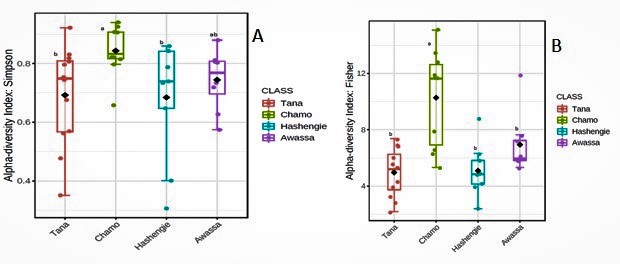

Supplement: Supplementary file 3 — Additional file 3. [file 12866_2022_2496_MOESM3_ESM.jpg]
