# Supplementary material for: The gut bacterial microbiome of Nile tilapia (Oreochromis niloticus) from lakes across an altitudinal gradient
Source: BMC Microbiol. 2022 Apr 4;22:87. doi: 10.1186/s12866-022-02496-z (PMC8978401; doi:10.1186/s12866-022-02496-z)
Supplement: Supplementary file 4 — Additional file 4: Supplementary Table 1. Relative abundance of microbial communities at the family level. Results are expressed as the mean ± standard error of mean (SEM). [file 12866_2022_2496_MOESM4_ESM.docx]

**Supplementary Table 1**: Relative abundance of microbial communities at the family level. Results are expressed as the mean ± standard error of mean (SEM).

| **Family** | **Mean±SEM** | | | |
| --- | --- | --- | --- | --- |
|  | Lake Awassa | Lake Chamo | Lake Hashengie | Lake Tana |
| Acetobacteraceae | 0.0001±0.0001 | 0.0044±0.0018 | 0.0007±0.0005 | 0 |
| Aeromonadaceae | 0.0000± | 0.0014±0.0009 | 0.0070±0.0043 | 0 |
| Bacillaceae | 0.0003±0.0002 | 0.0072±0.0024 | 0.0067±0.0051 | 0.0053±0.0035 |
| Barnesiellaceae | 0.0081±0.0073 | 0.0178±0.0107 | 0.0001±0.0001 | 0.0134±0.0042 |
| Beijerinckiaceae | 0.0049±0.0016 | 0.0022±0.0012 | 0.0092±0.0069 | 0.0013±0.0010 |
| Caldilineaceae | 0.0024±0.0010 | 0.0041±0.0019 | 0.0003±0.0002 | 0 |
| Clostridiaceae | 0.3930±0.0774 | 0.2413±0.0381 | 0.1657±0.0474 | 0.0777±0.0262 |
| Cyanobiaceae | 0.0072±0.0023 | 0.0001±0.0001 | 0 | 0.0006±0.0003 |
| Enterobacteriaceae | 0.0058±0.0056 | 0.0085±0.0034 | 0.0092±0.0048 | 0.0015±0.0005 |
| Erysipelotrichaceae | 0.1473±0.0293 | 0.0167±0.0047 | 0.0007±0.0004 | 0.0119±0.0062 |
| Fusobacteriaceae | 0.0155±0.0078 | 0.1456±0.0429 | 0.4752±0.1075 | 0.6692±0.0563 |
| Hyphomicrobiaceae | 0.0027±0.0013 | 0.0022±0.0008 | 0.0004±0.0004 | 0.0006±0.0005 |
| Kineosporiaceae | 0 | 0.0012±0.0004 | 0.0003±0.0003 | 0 |
| Lachnospiraceae | 0 | 0.0009±0.0004 | 0.0151±0.0096 | 0 |
| Legionellaceae | 0.0044±0.0020 | 0 | 0.0273±0.0156 | 0 |
| Methylococcaceae | 0.0031±0.0012 | 0.0363±0.0171 | 0.0016±0.0007 | 0 |
| Microbacteriaceae | 0 | 0 | 0.0021±0.0014 | 0 |
| Micrococcaceae | 0 | 0.0004±0.0003 | 0.0012±0.0007 | 0 |
| Microcystaceae | 0.0139±0.0032 | 0.0044±0.0022 | 0 | 0.0006±0.0003 |
| Microtrichaceae | 0.0018±0.0005 | 0 | 0 | 0 |
| Mycobacteriaceae | 0.0056±0.0023 | 0.0005±0.0003 | 0.0003±0.0003 | 0.0001±0.0001 |
| Nocardioidaceae | 0 | 0.0034±0.0015 | 0.0001±0.0001 | 0 |
| Oligoflexaceae | 0.0049±0.0024 | 0 | 0 | 0.0002±0.0002 |
| Peptostreptococcaceae | 0.3509±0.0661 | 0.3901±0.0688 | 0.2540±0.0809 | 0.1447±0.0197 |
| Rhizobiales_Incertae_Sedis | 0.0046±0.0036 | 0.0095±0.0042 | 0.0017±0.0011 | 0.0020±0.0011 |
| Rhodobacteraceae | 0 | 0.0065±0.0024 | 0.0001±0.0001 | 0 |
| Rickettsiaceae | 0.0030±0.0008 | 0.0021±0.0014 | 0 | 0.0009±0.0004 |
| Ruminococcaceae | 0.0025±0.0010 | 0 | 0 | 0 |
| SC_I_84 | 0 | 0.0041±0.0021 | 0 | 0.0004±0.0002 |
| Shewanellaceae | 0 | 0.0013±0.0007 | 0 | 0.0004±0.0003 |
| Silvanigrellaceae | 0 | 0 | 0 | 0.0351±0.0120 |
| Solirubrobacteraceae | 0 | 0 | 0.0037±0.0034 | 0 |
| Steroidobacteraceae | 0 | 0.0384±0.0198 | 0.0011±0.0007 | 0.0006±0.0003 |
| Sutterellaceae | 0.0001±0.0001 | 0.0102±0.0054 | 0.0001±0.0001 | 0.0003±0.0002 |
| Tannerellaceae | 0.0012±0.0012 | 0.0255±0.0134 | 0.0003±0.0003 | 0.0027±0.0011 |
| UBA12409 | 0.0025±0.0009 | 0 | 0 | 0 |
| uncultured | 0 | 0.0033±0.0014 | 0.0022±0.0021 | 0 |
| V19 | 0.0090±0.0030 | 0.0080±0.0029 | 0.0037±0.0027 | 0.0041±0.0026 |
| Vibrionaceae | 0.0052±0.0028 | 0.0022±0.0019 | 0.0101±0.0055 | 0.0265±0.0123 |
