# Supplementary material for: The gut bacterial microbiome of Nile tilapia (Oreochromis niloticus) from lakes across an altitudinal gradient
Source: BMC Microbiol. 2022 Apr 4;22:87. doi: 10.1186/s12866-022-02496-z (PMC8978401; doi:10.1186/s12866-022-02496-z)
Supplement: Supplementary file 5 — Additional file 5: Supplementary Table 2. Microbial communities identified at genus level from all samples. Results are expressed as the mean ± standard error of mean (SEM). [file 12866_2022_2496_MOESM5_ESM.docx]

**Supplementary Table 2**: Microbial communities identified at genus level from all samples. Results are expressed as the mean ± standard error of mean (SEM).

| **Genus** | **Mean±SEM** | | | |
| --- | --- | --- | --- | --- |
|  | Lake Awassa | Lake Chamo | Lake Hashengie | Lake Tana |
| Aeromonas | 0 | 0.0014±0.0009 | 0.0070±0.0043 | 0 |
| Aurantimicrobium | 0 | 0 | 0.0021±0.0014 | 0 |
| Bacillus | 0.0003±0.0002 | 0.0072±0.0024 | 0.0067±0.0051 | 0.0053±0.0035 |
| Candidatus_Megaira | 0 | 0.0021±0.0014 | 0 | 0.0007±0.0004 |
| Candidatus_Soleaferrea | 0.0025±0.0010 | 0 | 0 | 0 |
| Cetobacterium | 0.0124±0.0074 | 0.1355±0.0410 | 0.4750±0.1074 | 0.6628±0.0580 |
| Clostridium_sensu_stricto_1 | 0.1983±0.0622 | 0.0691±0.0143 | 0.1388±0.0362 | 0.0678±0.0231 |
| Clostridium_sensu_stricto_13 | 0 | 0.0112±0.0065 | 0.0008±0.0007 | 0.0025±0.0022 |
| Cyanobium_PCC_6307 | 0.0072±0.0023 | 0.0001±0.0001 | 0 | 0.0006±0.0003 |
| Epulopiscium | 0 | 0.0009±0.0004 | 0.0151±0.0096 | 0 |
| Hyphomicrobium | 0.0027±0.0013 | 0.0022±0.0008 | 0.0004±0.0004 | 0.0006±0.0005 |
| Legionella | 0.0022±0.0013 | 0 | 0.0243±0.0133 | 0 |
| Macellibacteroides | 0.0012±0.0012 | 0.0255±0.0134 | 0.0003±0.0003 | 0.0027±0.0011 |
| Methylocaldum | 0.0003±0.0002 | 0.0009±0.0005 | 0.0012±0.0007 | 0 |
| Methylocystis | 0.0003±0.0003 | 0.0004±0.0002 | 0.0092±0.0069 | 0 |
| Methyloparacoccus | 0.0015±0.0010 | 0.0039±0.0019 | 0.0004±0.0003 | 0 |
| Microcystis_PCC_7914 | 0.0139±0.0032 | 0.0044±0.0022 | 0 | 0.0006±0.0003 |
| Mycobacterium | 0.0056±0.0023 | 0.0005±0.0003 | 0.0003±0.0003 | 0.0001±0.0001 |
| Nocardioides | 0 | 0.0014±0.0006 | 0 | 0 |
| Plesiomonas | 0.0058±0.0056 | 0.0085±0.0034 | 0.0092±0.0048 | 0.0015±0.0005 |
| Romboutsia | 0.0006±0.0003 | 0.0852±0.0264 | 0.2132±0.0814 | 0.0230±0.0056 |
| Roseomonas | 0.0001±0.0001 | 0.0044±0.0018 | 0.0007±0.0005 | 0 |
| Shewanella | 0 | 0.0013±0.0007 | 0 | 0.0004±0.0003 |
| Silvanigrella | 0 | 0 | 0 | 0.0351±0.0120 |
| Turicibacter | 0.1473±0.0293 | 0.0167±0.0047 | 0.0007±0.0004 | 0.0119±0.0062 |
| Uncultured | 0.0253±0.0087 | 0.0800±0.0283 | 0.0063±0.0032 | 0.0166±0.0038 |
| V2 | 0.5725±0.0536 | 0.5370±0.0553 | 0.0885±0.0201 | 0.1679±0.0256 |
