# Supplementary material for: The gut bacterial microbiome of Nile tilapia (Oreochromis niloticus) from lakes across an altitudinal gradient
Source: BMC Microbiol. 2022 Apr 4;22:87. doi: 10.1186/s12866-022-02496-z (PMC8978401; doi:10.1186/s12866-022-02496-z)
Supplement: Supplementary file 6 — Additional file 6: Supplementary Table 3. Alpha diversity indexes and goods coverage of microbial 16S rRNA sequences from the gut of Nile tilapia collected from the four sampling lakes. [file 12866_2022_2496_MOESM6_ESM.docx]

**Supplementary Table 3**: Alpha diversity indexes and goods coverage of microbial 16S rRNA sequences from the gut of Nile tilapia collected from the four sampling lakes.

| No. | Lake | Samples | Observed | Chao 1 | ACE | Shannon | Simpson | Fisher | Goods |
| --- | --- | --- | --- | --- | --- | --- | --- | --- | --- |
| 1 | Tana | Au1CH | 27 | 33 | 39.038 | 1.439 | 0.562 | 5.323 | 99 |
| 2 | Tana | Au2CH | 13 | 23 | 19.078 | 0.796 | 0.351 | 2.181 | 99 |
| 3 | Tana | Au3CH | 25 | 25 | 25.326 | 2.092 | 0.796 | 4.838 | 100 |
| 4 | Tana | Au4CH | 16 | 17.5 | 18.741 | 1.613 | 0.676 | 2.801 | 100 |
| 5 | Tana | Au5CH | 24 | 26.143 | 27.854 | 1.392 | 0.568 | 4.600 | 99 |
| 6 | Tana | Au6CH | 35 | 37.625 | 41.672 | 2.395 | 0.833 | 7.369 | 99 |
| 7 | Tana | Au7CH | 27 | 42 | 41.938 | 2.064 | 0.818 | 5.323 | 99 |
| 8 | Tana | Au8CH | 36 | 41.143 | 43.229 | 2.217 | 0.805 | 7.636 | 99 |
| 9 | Tana | Au9CH | 34 | 35.5 | 35.939 | 2.879 | 0.925 | 7.104 | 100 |
| 10 | Tana | Au10CH | 22 | 27 | 30.148 | 1.928 | 0.754 | 4.132 | 99 |
| 11 | Tana | Au11CH | 18 | 20 | 21.670 | 1.168 | 0.477 | 3.232 | 100 |
| 12 | Tana | Au12CH | 29 | 32 | 36.161 | 1.959 | 0.744 | 5.819 | 99 |
| 13 | Chamo | C1CH | 61 | 66 | 66.704 | 3.282 | 0.939 | 15.093 | 99 |
| 14 | Chamo | C2CH | 51 | 54.273 | 56.308 | 2.543 | 0.822 | 11.937 | 99 |
| 15 | Chamo | C3CH | 56 | 59 | 61.621 | 3.117 | 0.927 | 13.486 | 99 |
| 16 | Chamo | C5CH | 49 | 53.5 | 55.591 | 2.919 | 0.908 | 11.333 | 99 |
| 17 | Chamo | C7CH | 35 | 54.5 | 50.799 | 1.696 | 0.658 | 7.369 | 98 |
| 18 | Chamo | C8CH | 27 | 27.857 | 30.300 | 2.156 | 0.812 | 5.323 | 100 |
| 19 | Chamo | C9CH | 55 | 61.429 | 60.833 | 2.686 | 0.839 | 13.172 | 99 |
| 20 | Chamo | C10CH | 32 | 41 | 40.570 | 2.108 | 0.796 | 6.582 | 99 |
| 21 | Chamo | C11CH | 31 | 40.333 | 37.899 | 2.182 | 0.823 | 6.325 | 99 |
| 22 | Chamo | C12CH | 52 | 63.143 | 61.899 | 2.849 | 0.906 | 12.242 | 98 |
| 23 | Hashengie | H1CH | 44 | 55 | 53.045 | 2.427 | 0.800 | 9.863 | 99 |
| 24 | Hashengie | H2CH | 30 | 33.75 | 34.353 | 2.272 | 0.844 | 6.071 | 99 |
| 25 | Hashengie | H3CH | 16 | 21 | 21.836 | 0.709 | 0.308 | 2.801 | 99 |
| 26 | Hashengie | H4CH | 26 | 28.143 | 31.243 | 2.204 | 0.859 | 5.079 | 99 |
| 27 | Hashengie | H5CH | 26 | 26.75 | 27.332 | 2.058 | 0.739 | 5.079 | 100 |
| 28 | Hashengie | H6CH | 24 | 24.6 | 25.972 | 1.764 | 0.648 | 4.600 | 100 |
| 29 | Hashengie | H7CH | 21 | 24 | 23.185 | 1.704 | 0.734 | 3.903 | 100 |
| 30 | Hashengie | H9CH | 22 | 25.333 | 25.707 | 1.074 | 0.401 | 4.132 | 99 |
| 31 | Hashengie | H10CH | 31 | 36.6 | 39.330 | 2.360 | 0.844 | 6.325 | 99 |
| 32 | Awassa | Aw4CH | 47 | 49.333 | 52.220 | 2.620 | 0.878 | 10.738 | 99 |
| 33 | Awassa | Aw5CH | 24 | 25.667 | 27.752 | 1.570 | 0.726 | 4.600 | 99 |
| 34 | Awassa | Aw6CH | 29 | 42.2 | 38.133 | 1.393 | 0.625 | 5.819 | 99 |
| 35 | Awassa | Aw8CH | 32 | 41.167 | 43.018 | 1.760 | 0.714 | 6.582 | 99 |
| 36 | Awassa | Aw9CH | 28 | 43 | 41.428 | 2.019 | 0.803 | 5.569 | 99 |
| 37 | Awassa | Aw10CH | 33 | 33.125 | 33.690 | 1.608 | 0.571 | 6.842 | 100 |
| 38 | Awassa | Aw11CH | 30 | 41 | 48.034 | 1.975 | 0.811 | 6.071 | 99 |
| 39 | Awassa | Aw12CH | 27 | 31 | 37.102 | 1.933 | 0.804 | 5.323 | 99 |
