# Supplementary material for: The gut bacterial microbiome of Nile tilapia (Oreochromis niloticus) from lakes across an altitudinal gradient
Source: BMC Microbiol. 2022 Apr 4;22:87. doi: 10.1186/s12866-022-02496-z (PMC8978401; doi:10.1186/s12866-022-02496-z)
Supplement: Supplementary file 7 — Additional file 7: Supplementary Table 4. Spearman correlation between the relative abundances of gut microbial communities at genus level and altitude. **. Correlation is significant at the 0.01 level (2-tailed). *. Correlation is significant at the 0.05 level (2-tailed). [file 12866_2022_2496_MOESM7_ESM.docx]

**Supplementary Table 4**: Spearman correlation between the relative abundances of gut microbial communities at genus level and altitude. **. Correlation is significant at the 0.01 level (2-tailed). *. Correlation is significant at the 0.05 level (2-tailed).

| Taxa | Correlation Coefficient | Sig. (2-tailed) |
| --- | --- | --- |
| Aeromonas | 0.196 | 0.233 |
| Aurantimicrobium | 0.398* | 0.012 |
| Bacillus | -0.166 | 0.313 |
| Candidatus_Megaira | -0.214 | 0.190 |
| Candidatus_Soleaferrea | -0.171 | 0.299 |
| Cetobacterium | 0.576** | 0.000 |
| Clostridium_sensu_stricto_1 | 0.042 | 0.802 |
| Clostridium_sensu_stricto_13 | -0.380* | 0.017 |
| Cyanobium_PCC_6307 | -0.199 | 0.224 |
| Epulopiscium | 0.267 | 0.100 |
| Hyphomicrobium | -0.409** | 0.010 |
| Legionella | 0.570** | 0.000 |
| Macellibacteroides | -0.380* | 0.017 |
| Methylocaldum | -0.042 | 0.801 |
| Methylocystis | 0.259 | 0.111 |
| Methyloparacoccus | -0.479** | 0.002 |
| Microcystis_PCC_7914 | -0.499** | 0.001 |
| Mycobacterium | -0.285 | 0.079 |
| Nocardioides | -0.510** | 0.001 |
| Plesiomonas | -0.039 | 0.813 |
| Romboutsia | 0.192 | 0.242 |
| Roseomonas | -0.370* | 0.020 |
| Shewanella | -0.319* | 0.048 |
| Silvanigrella | 0.208 | 0.203 |
| Turicibacter | -0.604** | 0.000 |
| Uncultured | -0.494** | 0.001 |
| V2 | -0.788** | 0.000 |
