# Supplementary material for: The gut bacterial microbiome of Nile tilapia (Oreochromis niloticus) from lakes across an altitudinal gradient
Source: BMC Microbiol. 2022 Apr 4;22:87. doi: 10.1186/s12866-022-02496-z (PMC8978401; doi:10.1186/s12866-022-02496-z)
Supplement: Supplementary file 8 — Additional file 8: Supplementary Table 5. Microbial communities showing a significant difference at family level between sampling lakes. Comparison was done by independent t-test. [file 12866_2022_2496_MOESM8_ESM.docx]

**Supplementary Table 5**: Microbial communities showing a significant difference at family level between sampling lakes. Comparison was done by independent t-test.

| **Families** | **t-test for Equality of Means** | | | | |
| --- | --- | --- | --- | --- | --- |
|  | **Between Lake Tana and Lake Awassa** | | **Between Lake Tana and Lake Chamo** | | |
|  | **t** | **Sig. (2-tailed)** | **Families** | **t** | **Sig. (2-tailed)** |
| Acetobacteraceae | -1.24 | 0.23 | Acetobacteraceae | -2.96* | 0.01 |
| Bacillaceae | 0.96 | 0.35 | Aeromonadaceae | -1.74 | 0.10 |
| Barnesiellaceae | 0.76 | 0.46 | Bacillaceae | -0.56 | 0.58 |
| Beijerinckiaceae | -2.17* | 0.04 | Barnesiellaceae | -0.45 | 0.65 |
| Caldilineaceae | -2.41* | 0.03 | Beijerinckiaceae | -1.02 | 0.32 |
| Clostridiaceae | -4.31* | 0.00 | Caldilineaceae | -2.45* | 0.02 |
| Comamonadaceae | 0.81 | 0.43 | Clostridiaceae | -3.53* | 0.00 |
| Cyanobiaceae | -3.55* | 0.00 | Comamonadaceae | -2.77* | 0.01 |
| Enterobacteriaceae | -0.79 | 0.44 | Cyanobiaceae | 0.91 | 0.37 |
| Erysipelotrichaceae | -5.45* | 0.00 | Enterobacteriaceae | -1.95 | 0.07 |
| Fusobacteriaceae | 9.49* | 0.00 | Erysipelotrichaceae | -0.32 | 0.75 |
| Hyphomicrobiaceae | -1.63 | 0.12 | Fusobacteriaceae | 7.27* | 0.00 |
| Lachnospiraceae | -1.24 | 0.23 | Hyphomicrobiaceae | -0.76 | 0.46 |
| Legionellaceae | -2.02 | 0.06 | Kineosporiaceae | -3.09* | 0.01 |
| Methylococcaceae | -4.07* | 0.00 | Lachnospiraceae | -2.48* | 0.02 |
| Methyloligellaceae | 1.09 | 0.29 | Legionellaceae | -0.57 | 0.57 |
| Microcystaceae | -5.38* | 0.00 | Methylococcaceae | -2.48* | 0.02 |
| Microtrichaceae | -3.80* | 0.00 | Methyloligellaceae | -0.03 | 0.98 |
| Mycobacteriaceae | -3.88* | 0.00 | Micrococcaceae | -1.55 | 0.14 |
| Oligoflexaceae | -2.22* | 0.04 | Microcystaceae | -2.09 | 0.05 |
| Peptostreptococcaceae | -3.57* | 0.00 | Microtrichaceae | 0.91 | 0.37 |
| Rhizobiales_Incertae_Sedis | -0.71 | 0.48 | Mycobacteriaceae | -3.30* | 0.00 |
| Rhodobacteraceae | 0.81 | 0.43 | Nocardioidaceae | -2.38* | 0.03 |
| Rickettsiaceae | -1.54 | 0.14 | Peptostreptococcaceae | -3.79* | 0.00 |
| Ruminococcaceae | -2.15 | 0.05 | Rhizobiales_Incertae_Sedis | -1.60 | 0.13 |
| SC_I_84 | -1.24 | 0.23 | Rhodobacteraceae | -2.37* | 0.03 |
| Silvanigrellaceae | 2.31* | 0.03 | Rickettsiaceae | -0.93 | 0.36 |
| Steroidobacteraceae | 1.64 | 0.12 | SC_I_84 | -2.35* | 0.03 |
| Sutterellaceae | 0.24 | 0.81 | Shewanellaceae | -1.75 | 0.09 |
| Tannerellaceae | 2.06 | 0.05 | Silvanigrellaceae | 2.60* | 0.02 |
| V19 | -2.88* | 0.01 | Steroidobacteraceae | -1.92 | 0.07 |
| Vibrionaceae | 1.42 | 0.17 | Sutterellaceae | -2.36* | 0.03 |
|  |  |  | Tannerellaceae | -1.80 | 0.09 |
|  |  |  | uncultured | -2.87* | 0.01 |
|  |  |  | V19 | -1.32 | 0.20 |
|  |  |  | Vibrionaceae | 1.90 | 0.07 |
